# Regulation of *Trypanosoma brucei* Total and Polysomal mRNA during Development within Its Mammalian Host

**DOI:** 10.1371/journal.pone.0067069

**Published:** 2013-06-26

**Authors:** Paul Capewell, Stephanie Monk, Alasdair Ivens, Paula MacGregor, Katelyn Fenn, Pegine Walrad, Frederic Bringaud, Terry K. Smith, Keith R. Matthews

**Affiliations:** 1 Centre for Immunity, Infection and Evolution, University of Edinburgh, Edinburgh, United Kingdom; 2 Biomedical Sciences Research Complex, University of St Andrews, St Andrews, United Kingdom; 3 Centre de Résonance Magnétique des Systèmes Biologiques (RMSB), Université Bordeaux Segalen, Bordeaux, France; Instituto Butantan, Laboratório Especial de Toxinologia Aplicada, Brazil

## Abstract

The gene expression of *Trypanosoma brucei* has been examined extensively in the blood of mammalian hosts and in forms found in the midgut of its arthropod vector, the tsetse fly. However, trypanosomes also undergo development within the mammalian bloodstream as they progress from morphologically ‘slender forms’ to transmissible ‘stumpy forms’ through morphological intermediates. This transition is temporally progressive within the first wave of parasitaemia such that gene expression can be monitored in relatively pure slender and stumpy populations as well as during the progression between these extremes. The development also represents the progression of cells from translationally active forms adapted for proliferation in the host to translationally quiescent forms, adapted for transmission. We have used metabolic labelling to quantitate translational activity in slender forms, stumpy forms and in forms undergoing early differentiation to procyclic forms *in vitro*. Thereafter we have examined the cohort of total mRNAs that are enriched throughout development in the mammalian bloodstream (slender, intermediate and stumpy forms), irrespective of strain, revealing those that exhibit consistent developmental regulation rather than sample specific changes. Transcripts that cosediment with polysomes in stumpy forms and slender forms have also been enriched to identify transcripts that escape translational repression prior to transmission. Combined, the expression and polysomal association of transcripts as trypanosomes undergo development in the mammalian bloodstream have been defined, providing a resource for trypanosome researchers. This facilitates the identification of those that undergo developmental regulation in the bloodstream and therefore those likely to have a role in the survival and capacity for transmission of stumpy forms.

## Introduction

Like many protozoan pathogens, African trypanosomes undergo a complex life cycle requiring passage between mammalian hosts by an arthropod vector [1], [2]. Specifically, the parasite is transmitted by tsetse flies, blood-feeding dipterans that ingest circulating trypanosome parasites in the bloodstream of an infected mammalian host. For successful colonisation of the tsetse midgut specialised ‘stumpy’ form transmission stages must be ingested by the tsetse fly [3], [4], [5]. Stumpy forms are characterised by their division arrest and increased resistance to stresses associated with transmission, such as changes in temperature, pH and proteolytic environment [6]. Stumpy forms are also adapted for transmission in their ability to detect the differentiation signals citrate and cis-aconitate [7], [8], [9] due to their expression of members of the PAD family, an array of carboxylate transporter proteins specifically expressed on stumpy and tsetse midgut procyclic forms [10].

The development of stumpy forms in the mammalian bloodstream appears to be regulated by a density sensing mechanism [11], [12], [13]. As parasite numbers accumulate in the bloodstream of a mammalian host through the division of proliferative slender forms, a putative signal that acts as a stumpy induction factor (SIF) is proposed to accumulate [11]. This factor (or factors) is detected by the parasites in the circulation and once sufficient signal accumulates, cell cycle arrest is stimulated, leading to the development of stumpy forms. Although the molecular details of stumpy formation are not precisely characterised, differentiation follows a temporally predictable procession of events, involving down regulation of the expression of the parasite haptoglobin-heamoglobin receptor [14], cell cycle arrest [15], activation of the expression of ESAG9 proteins [16], [17] and the expression of PAD mRNAs [10], [18], [19]. These events are followed by the morphological transformation of parasites to stumpy forms from intermediate forms. Such changes involve the elaboration of the parasite mitochondrion (in preparation for the switch to a mitochondrial based respiration in the tsetse mid-gut [20]), expansion of the parasite’s lysosome and flagellar pocket [14] and migration of the nucleus toward the posterior end of the cell. Nuclear migration can be such that the terminal kinetoplast can be displaced leading to a postero-nuclear phenotype [5].

Although the signals underlying the stimulation of stumpy formation are unknown, quantitative analysis of the production and maintenance of stumpy forms in a chronic bloodstream form infection has allowed modelling of several key parameters [19]. For example, the bloodstream trypanosome population is predicted to be composed of uncommitted slender cells (i.e. those that will continue to replicate indefinitely), committed slender cells (those that have detected the SIF signal and are undergoing limited further divisions before cell cycle arrest), intermediate forms (that are irreversibly division arrested but have not yet undergone morphological transformation to stumpy forms) and stumpy forms themselves. From analyses of the development of the first wave of parasitaemia, and further analysis of the proportion of stumpy cells in long term established infections, only slender cells (whether committed or uncommitted) were predicted to produce SIF. Cells that become committed were predicted to undergo around three cell divisions prior to irreversible arrest as intermediate forms, which then differentiate over 48 h to produce morphologically stumpy forms with a lifespan of a further 48–72 h. The consequence of this temporal programme is that intermediate and stumpy forms comprise the majority of a chronic bloodstream infection and trypanosome infections are therefore optimised for transmission [5]. This is an important consideration as most molecular analyses of trypanosomes focus on the proliferative slender forms but in the context of chronic infection, intermediate and stumpy forms may represent the dominant cell types.

In the experiments described here we have analysed gene expression in the different developmental forms that characterise natural trypanosome infections in the mammalian bloodstream. In kinetoplastid parasites, the organisation of the genome into polycistronic arrays necessitates that regulated gene expression is overwhelmingly governed by post-transcriptional mechanisms, particularly mRNA abundance [21]. However, the importance of translational control is also increasingly recognised as a key regulatory step, such that mRNA abundance and the expression of gene protein products do not always correlate in eukaryotes [22], [23], [24]. This is particularly evident in the generation of stumpy forms, where the global translation of trypanosome genes has been observed to be considerably reduced with respect to both bloodstream slender and procyclic forms [25]. In order to examine such regulatory events we have carried out analyses of two processes associated with trypanosome infection. Firstly, we have examined the progressive changes in gene expression that accompany the transition from slender forms, through intermediate forms, to stumpy forms in two distinct trypanosome strains. This identified mRNAs that show regulatory trends through development, aiding the ability to separate developmental changes in RNA from sample specific or procedural specific changes, such as changes associated with cell isolation procedures. Secondly, we have analysed those mRNAs that co-sediment with polysomal material in either slender or stumpy forms. These polysomally-enriched transcripts are more likely to be actively translated, or poised ready for translation, and will reveal transcripts in stumpy forms that actively escape global translational repression. These are likely to be important in transmission processes, such as during establishment in the tsetse fly. Combined, these data sets provide a resource for understanding how trypanosomes develop in the mammalian bloodstream in order to ensure their successful passage to their tsetse vector.

## Results

### Analysis of Translation During Trypanosoma Brucei Development

**Figure 1:**
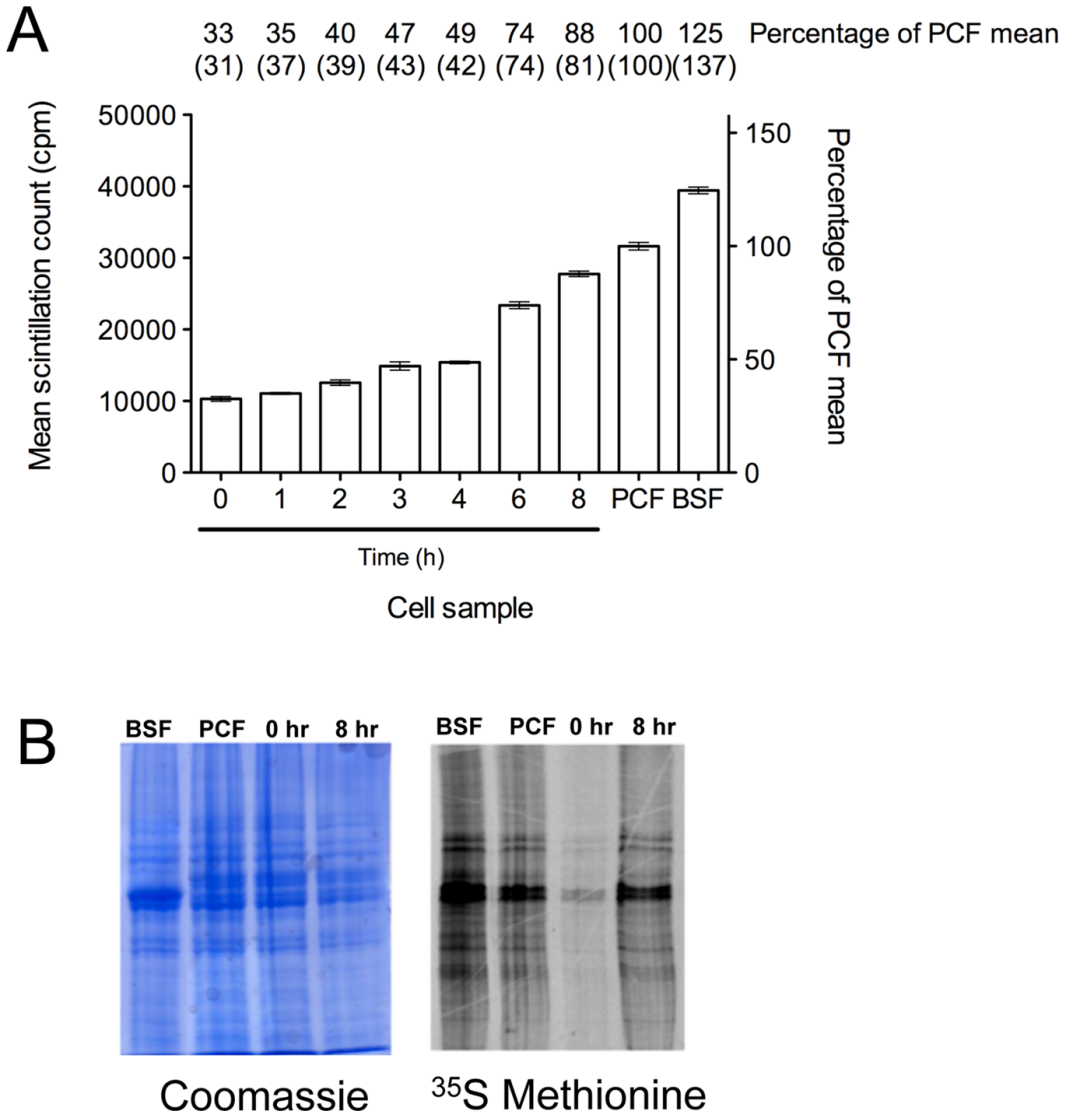
Escape from translational repression during differentiation from bloodstream to procyclic forms. A. Mean scintillation counts ± standard error of [^35^S]-methionine incorporation during 1 hour windows throughout the differentiation from stumpy forms to procyclic forms (n = 3). Samples were taken at various time-points, shown in hours (h), post-addition of *cis*-aconitate (6mM); 0 h represents stumpy form cells. Established procyclic form cells (427 449 strain; PCF) and slender bloodstream monomorphic cells (single marker strain; BSF) were also analysed for comparison. Shown on the right *y*-axis is the mean count as a percentage of the mean count for established procyclic form cells (100%), these values also being shown above each data point. The experiment was performed twice and the numbers in parentheses are those obtained from the second experiment. B. Visualization of protein synthesis throughout differentiation by [^35^S]-methionine labelling. 6mM *cis*-aconitate was added to stumpy form *T. b. brucei* AnTat1.1 cells; samples were then labelled with [^35^S]-methionine for one hour at the time indicated post-addition of cis-aconitate (hr). The 0 hr time-point represents stumpy form cells. Monomorphic slender bloodstream form (BSF) and established procyclic form (PCF) cultures were also analysed. Protein samples were separated by SDS-PAGE (10%) and stained with Coomassie (left hand panel) to show loading or assayed by fluorography (right hand panel) to detect [^35^S]-labelled proteins. The major labelled bands are tubulin and VSG (for Slender forms), which closely migrate. Stumpy forms show less obvious VSG since MSP-B and GPI-PLC activity during parasite isolation and pre-incubation before labelling resulted in a significant loss of cell-associated VSG.

The relative translation of slender and stumpy forms has been previously studied by analysis of their polysome profiles, indicating that stumpy forms exhibit reduced protein synthesis [25]. In order to confirm this and to determine when translational repression becomes relieved during the differentiation to procyclic forms, the rate of radiolabelled methionine incorporation into *de novo* protein synthesis was determined in slender and stumpy forms as well as during the *in vitro* differentiation between stumpy and procyclic forms. Figure 1A shows that the ^35^S methionine incorporation in slender and stumpy forms was 125% and 33%, relative to cultured procyclic forms (normalised to 100%), respectively. During *in vitro* differentiation of stumpy to procyclic forms, the rate of ^35^S methionine incorporation remained low for the first four hours of differentiation (33–49% of procyclic form levels) then rapidly increased to 88% of procyclic levels between 4 and 8 hours. Although there was a strong increase in label incorporation into protein, analysis by SDS PAGE did not resolve major discrete bands showing an ‘immediate early’ synthesis; between 0 and 8 hours the resolved profile was not detectably different (the profile of labelled proteins at 0h and 8h is shown in Figure 1B). These kinetics of protein synthesis coincide with the reported increase in cellular RNA associated with differentiation to procyclic forms [26].

### mRNA Profile of Cells Undergoing Development in the Mammalian Blood

In order to determine the changes in mRNA expression profile between slender, intermediate and stumpy forms, RNAs were prepared from each life cycle stage harvested 3 days (slender), 4–5 days (intermediate) or 6–7 (stumpy) days after the initiation of infections with *T. brucei* strains AnTat.1.1 or EATRO 2340. These two independent strains follow similar developmental kinetics and biological characteristics during the slender to stumpy transition and were used to aid the identification of mRNAs whose abundance changed consistently upon development from slender to stumpy forms irrespective of parasite strain. This was an important consideration given the variation in transcript levels observed in different studies of developmental gene expression in trypanosomes [17], [27], [28]. Moreover, intermediate forms were analysed for the first time to assist the identification of transcripts that showed progressive increase or decrease in abundance between slender and stumpy forms, or transient increase at the intermediate stage. These profiles were expected to highlight programmed developmental changes.

Total RNA from each life cycle stage of these two strains was polyA-enriched, converted to cDNA, and sequenced using Digital SAGE transcriptome techniques. Although this approach has been largely superseded by RNAseq methods since the experiments were carried out, Digital SAGE provides information of similar sensitivity with respect to relative mRNA abundance and at lower cost, enabling more biological samples to be analysed. RNA samples were prepared as previously described [29], fragmented using *Nla*III and Solexa sequenced at MWG Eurofins Operon and ‘The Genepool’, University of Edinburgh (library coverage data for each sample is provided in Table S1). Adapter sequences and short or poor quality reads were trimmed from the data using Trimmomatic and aligned using SMALT v0.6.4 with a maximum of 2 mismatches per read and multiple mappings given a score of 0. The alignment index for SMALT was created with a word length of 11 from the most recent GeneDB trypanosome contigs (version: 13/03/2012) (http://www.genedb.org/Homepage/Tbruceibrucei927) with ORFs extended using the predicted UTR data from the Cross Laboratory [30]. Including the predicted UTRs increased the ability to map alignment reads to whole transcripts through its inclusion of more possible *Nla*III fragments [31]. Pairwise comparisons of the relative expression of transcripts between life cycle stages were performed using the DESeq 1.11.2 package within R 2.15.1 using a binomial model and a blind variance stabilising transformation, this normalising for variation in raw read counts from individual samples. A generous p-value cut-off of 0.1 was used to support the differential expression of transcripts. It should be noted that the number of replicates used did not allow a robust analysis of false discovery rate so experimental sampling was also used to support the expression profile of transcripts likely to show developmental regulation.

### Developmental Regulation of mRNA Families: Pathway Analysis

**Figure 2:**
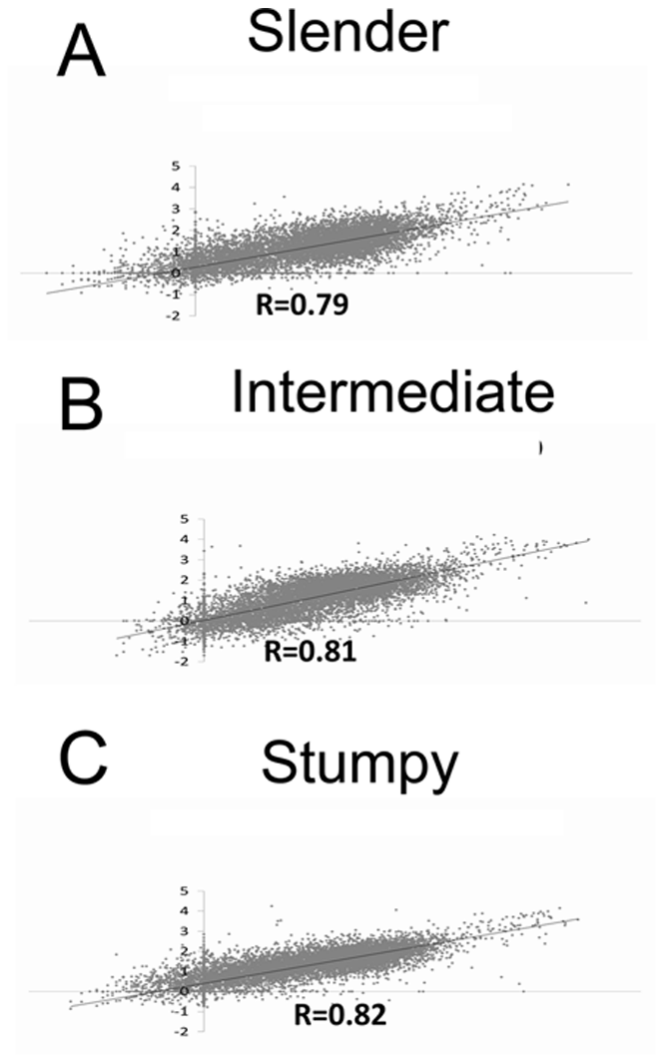
Comparison of the relative expression of genes in different strains of T. brucei at different life cycle stages. Scatter plots of mRNA abundance are shown of Slender (Panel A), Intermediate (Panel B) and Stumpy forms (panel C) using material derived from *T. brucei* AnTat1.1 (x axis) and *T. brucei* EATRO (y axis) parasites.

Comparison between the two strains of *T. brucei* either as slender, intermediate or stumpy forms revealed similar expression profiles, with correlation coefficient R^2^ values of 0.81, 0.78 and 0.83 respectively (Figure 2). Pair-wise comparisons between the life cycle stages identified several transcripts that differ between life cycle stages (*Data File S1*; for completeness all transcripts are included regardless of the inter-strain variance). The mRNA profiles of intermediate and stumpy forms were almost identical with a correlation coefficient R^2^ of 0.97, highlighting the fact that cells have committed to transmission and quiescence long before morphological changes are apparent. Both intermediate and stumpy form transcript profiles correlate somewhat with slender forms, but to a lesser degree (R^2^ of 0.76 for stumpy cells, R^2^ of 0.82 for intermediates). This analysis identified 61 transcripts whose abundance was ≥2 fold in stumpy forms compared to slender forms and 43 were ≥3 fold more abundant (*Data File* S2). Of those transcripts showing an abundance of ≥2 fold, 29 (48%) were already elevated in intermediate forms with respect to slender forms, again confirming that many changes in transcript abundance occur early in the development to stumpy forms. Those elevated in intermediate forms (with respect to fold change in comparison to slender forms) (*Data File S*3) included PSSA-2 (*Tb*927.10.11220) and MAPK2 (*Tb*927.10.16030), the latter being involved in the proliferation of procyclic form parasites upon differentiation in the tsetse mid-gut [32]. Several nucleolar proteins were also elevated including NOPP44 (*Tb*927.8.760), Fibrillarin (*Tb*927.10.14750), Gu (*Tb*927.5.4420), cyclophilin NCP1(*Tb*927.8.2000) and Puf7 (*Tb*11.01.6600), the latter two of which have been shown to interact with each other [33]. Among the stumpy-enriched transcripts were several that affect transmission to the tsetse stage of the infection. These include MSP-B (*Tb*927.8.1640), required for VSG release upon differentiation, PSSA-2 (*Tb*927.10.11220) and *Tb*PIP39 (*Tb*09.160.4460), required for recognition of the CCA differentiation signal [34]. In common with intermediate cells, there are nucleolar-associated transcripts that are enriched that may be involved in quiescence (NOG1 *Tb*11.02.0620). Reassuringly, several transcripts previously identified as being elevated in stumpy forms are present in our dataset. These include the purine nucleoside transporter NT10 (*Tb*09.160.5480) [35] and the previously mentioned *Tb*PIP39, as well as most of the cohort of stumpy enriched mRNAs detected by Nimblegen analysis [17]. PAD genes [10] were not strongly represented because the similarity between family members prevented the unique assignment of reads to specific members, although PAD2 could be identified as enriched based on its discriminatory long 3′UTR. Of the most enriched 61 stumpy transcripts, 39 were annotated as being hypothetical proteins of unknown function (although closer inspection suggests that many have a mitochondrial location prediction). Although formal studies elucidating the function of these genes will be required, targeted RNAi of 9 of these transcripts by a genome-wide approach generated defects in differentiation, with a further 3 severely limiting viability of procyclic forms [36].

**Figure 3:**
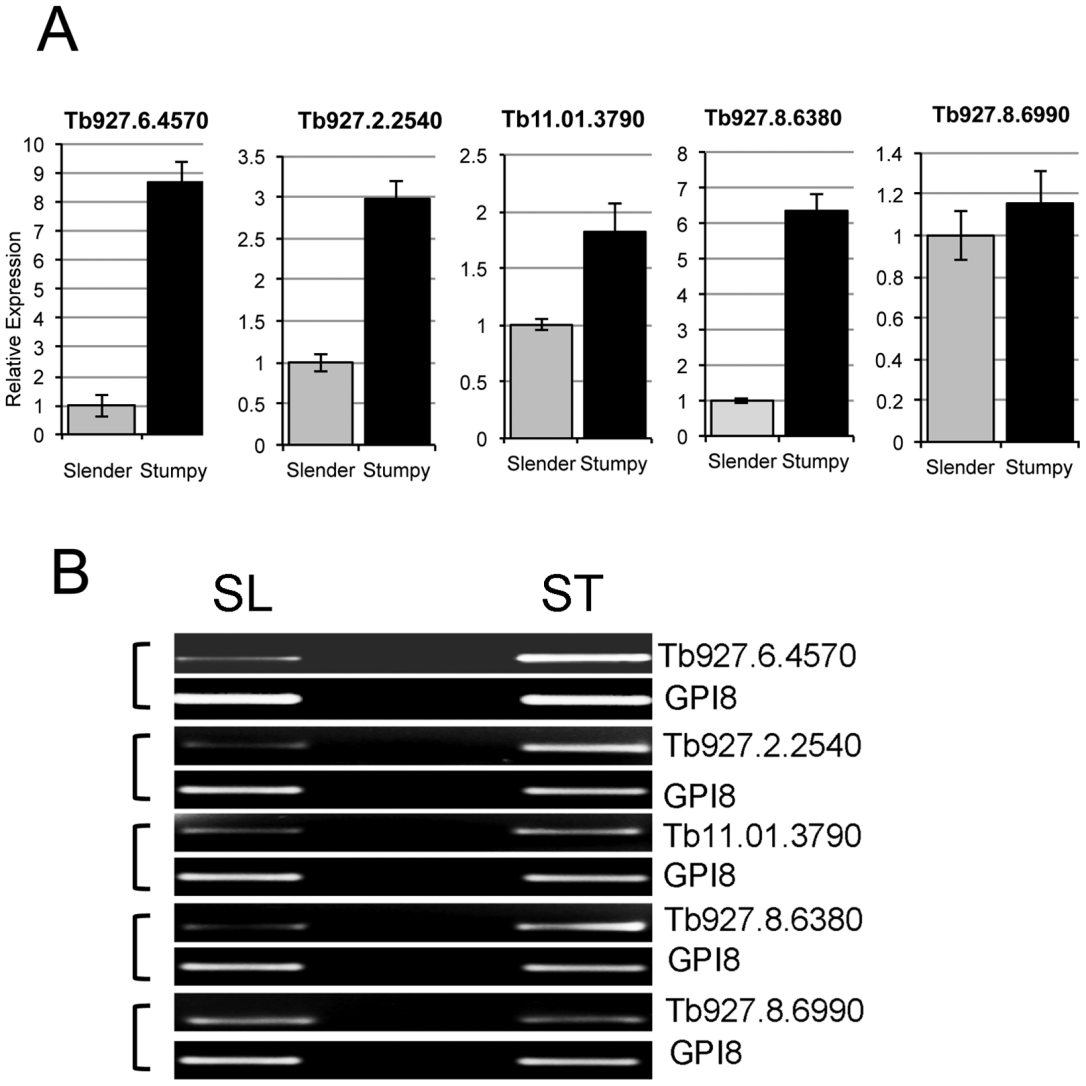
Validation of expression profiles detected by the transcriptome study. A. Realtime qRT PCR data for the relative expression of five transcripts predicted to be enriched in stumpy forms. mRNAs from slender and stumpy forms were subject to real time qRT-PCR using a constitutive transcript, GPI8, as a normalisation control. Although the scale of relative expression in each case differed from the global transcriptome study the direction of change, and the enrichment of each transcript in stumpy forms, was consistent, with the exception of Tb927.8.6990. B. qRT PCR products of the test transcripts in panel A, validating differential expression. In each case GPI8, which does not show statistically significant variation between life cycle stages, provided the normalisation control [58].

**Figure 4:**
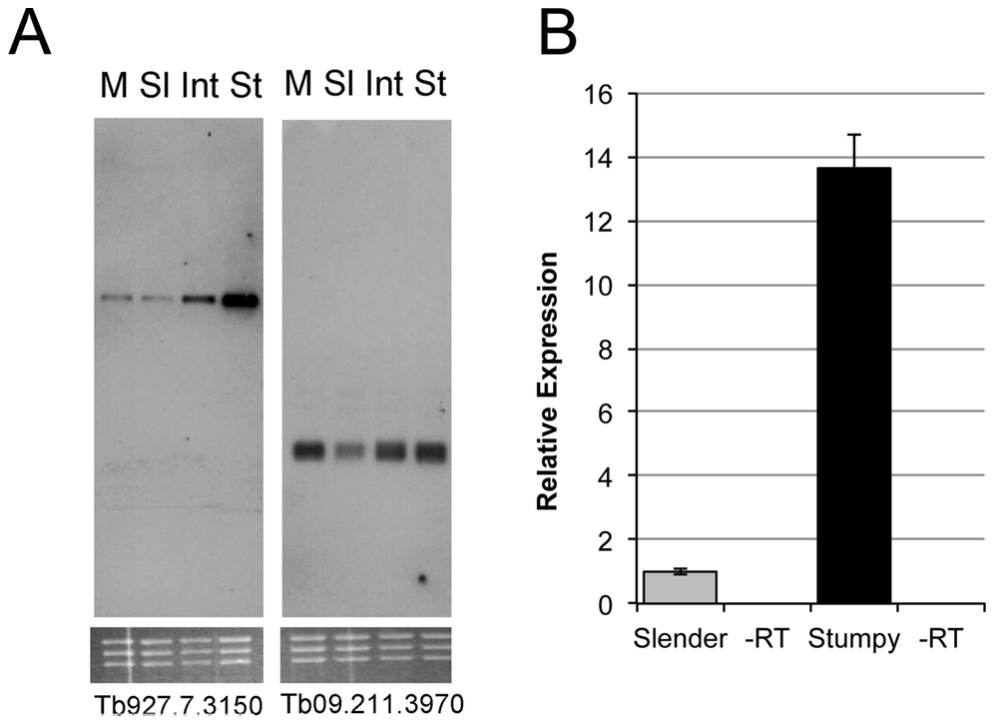
Expression profile of Tb927.7.3150. A. Northern blot validation of the developmental expression of transcript *Tb*927.7.3150. RNA from monomorphic slender (M) and pleomorphic slender (Sl), intermediate (Int) and stumpy (St) forms of *T. brucei* AnTat 1.1 was hybridized with riboprobes specific for either *Tb*927.7.3150 (left hand panel) or a control transcript *Tb*09.211.3970. The expression of *Tb*927.7.3150 progressively increases from slender to intermediate, being highest in stumpy forms. B. Realtime qRTPCR of the relative expression of *Tb*927.7.3150 in slender and stumpy forms of *T. brucei* AnTat 1.1. Transcript *Tb*927.10.13860 (GPI8) provided the normalisation control.

As false discovery rate (FDR) could not be robustly estimated for the dataset, five genes predicted to be enriched in stumpy cells were selected for verification by qRT PCR (*Tb*927.6.4570; *Tb*927.2.2540; *Tb*11.01.3790; *Tb*927.8.6380; *Tb*927.8.6990; *Tb*927.7.3150) (Figure 3, Figure 4B). All of these transcripts showed significantly increased abundance in stumpy form RNA relative to slender form RNA (*Tb*927.6.4570, p = 0.036; *Tb*927.2.2540, p = 0.035; *Tb*11.01.3790, p = 0.036; *Tb*927.8.6380, p = 0.019; *Tb*927.7.3150, p<0.001), except *Tb*927.8.6990 (p = 0.284). *Tb*927.7.3150, encoding a protein with a predicted CRAL-TRIO domain (see later) was also analysed by Northern blotting (Figure 4A) and this again showed the expected progressive increase in expression from slender to intermediate and stumpy forms. A control transcript predicted not to be significantly regulated did not show this pattern (*Tb*09.211.3970) (Figure 4A).

These analyses identified expression changes likely to reflect the adaptation of the parasites for differentiation to procyclic forms upon transmission to tsetse flies. To analyse the extent of adaptation in more detail two further analyses were carried out: (1) analyses of transcripts associated with metabolic pathways and (2) analysis of the differential expression of the predicted surface phylome.

### Developmental Regulation of mRNA Families: Metabolic Pathway Analysis

Pathway analysis was carried out using an iterative group analysis, a method that identifies changes in components of pathways, rather than averaging the changes across an entire pathway [37]. Pathways that were significantly (P<0.01) up-regulated or down-regulated in stumpy forms with respect to slender forms are shown in Table 1, with the number of changed components of the pathway being indicated (full expression profile analyses for different metabolic pathways is provided in *Data File S*4). This confirmed the expected elevation of the glycolytic pathway in slender forms with respect to stumpy forms, whereas gene expression for components of the TCA cycle (10/16 genes changed) and respiratory chain (71/80 members) was elevated in stumpy forms, consistent with their preadaptation for differentiation to tsetse mid-gut forms. Other changes in the transcriptome generally matched the expected trend, although there was considerable variation between samples for many enzymes reducing confidence in their expression profile. This might reflect differences in the history of the stumpy form populations (i.e. time since stumpy formation) such that the elevation of a transcript in preparation for transmission might be counteracted by its decay as the stumpy cells age.

**Table 1:** Pathways whose components exhibit elevated, or decreased, expression in stumpy forms.

Change	Process	Members	Number changed	% Changed
**Up in Stumpy**	Respiratory chain	80	71	89
**Up in Stumpy**	Mitochondrial carrier	25	12	48
**Up in Stumpy**	TCA cycle	16	10	62
**Down in Stumpy**	Sphingolipid Biosynthesis	11	11	100
**Down in Stumpy**	Fatty Acid Biosynthesis	13	8	62
**Down in Stumpy**	Glycolysis	43	19	44
**Down in Stumpy**	Proline and Glutamine Metabolism	8	3	38

### Developmental Regulation of mRNA Families: Surface Phylome Analysis

**Figure 5:**
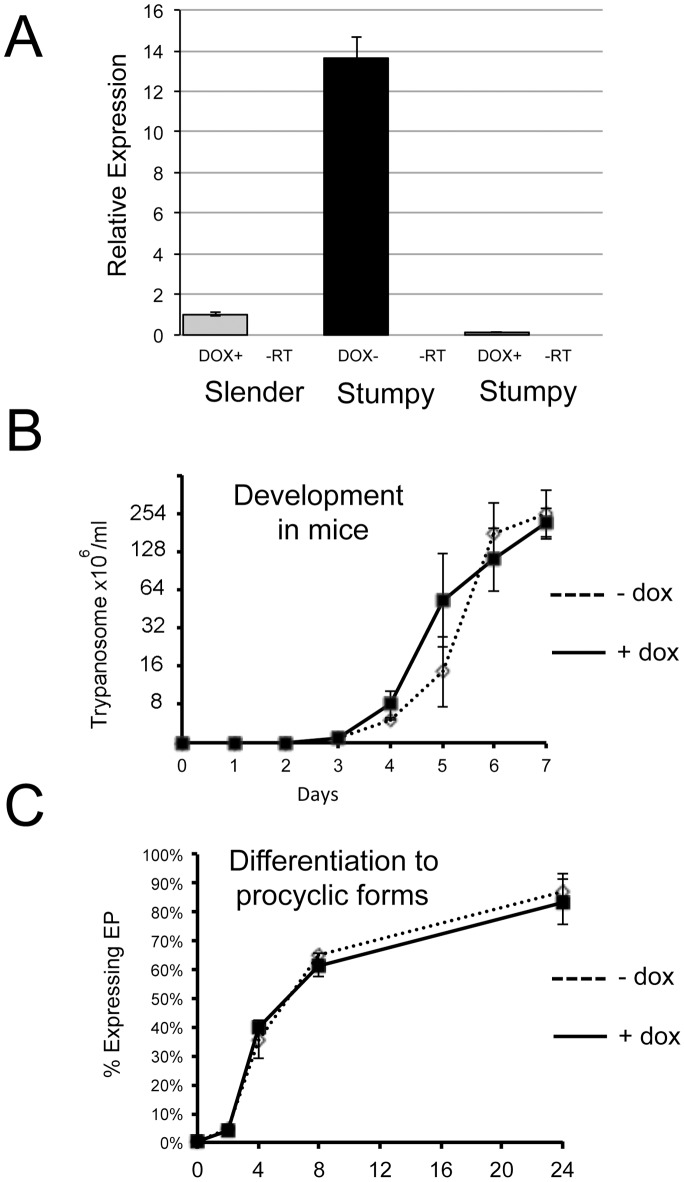
RNA interference against Tb927.7.3150 does not perturb differentiation *in vivo*. A. Realtime qRT PCR analysis of the expression and RNAi mediated knockdown of *Tb*927.7.3150 mRNA in pleomorphic *T. brucei* AnTat1.1 90:13 cells. Samples were derived from rodent infections with RNAi being induced by doxycycline provided in the drinking water. RNAi induction generated depletion of the *Tb*927.7.3150 transcripts to levels below that detected in slender forms. B. Progression of the course of parasitaemia of *Tb*927.7.3150 RNAi lines induced (solid lines), or not (dashed lines), to deplete the target transcript. No difference in the progression of the parasitaemia was observed whether RNAi was induced or not. n = 3. C. *Tb*927.7.3150 parasites either induced (solid lines), or not (dashed lines), were harvested from rodent infections once predominantly stumpy in morphology (on day 7 post infection for both induced and uninduced samples). Their differentiation to procyclic forms was then monitored by the expression of EP procyclin. In both cases the expression of this differentiation marker was equivalent regardless of the knockdown of *Tb*927.7.3150 mRNA levels.

In addition to this pathway analysis, mRNAs enriched in stumpy forms were compared against the recently published surface phylome of trypanosomatid species [38], in order to identify any transmission-stage enriched genes that may encode proteins expressed at the parasite surface. Hence genes at least two fold enriched in stumpy forms were analysed with respect to their surface phylome category (*Data File S*5). As described above, the gp63 family MSP-B (Phylome family 46), purine nucleoside transporters (Family 61; including NT10 [35] and NT8.1), and PAD2 and PAD3 (family 58) [10] were each up-regulated in stumpy forms as were a number of amino acid transporters (family 54), trans-sialidases (family 47) and a magnesium transporter protein (family 55). The latter proteins are also elevated in *Leishmania* parasites where their expression is associated with the transition to amastigotes forms [39]. Of the *T. brucei* specific members, only one of the b-type VSG family was enriched. Several hypothetical proteins were also elevated in stumpy forms including a protein of unknown function with no identifying domains (*Tb*927.7.6110) and one Family 80 member, *Tb*927.7.3150, with a characteristic CRAL-TRIO domain (Northern blots for this transcript were shown in Figure 4A). CRAL/TRIO domains bind small lipophilic molecules and proteins bearing such domains are involved in actin remodelling and phospholipid transfer. Whilst qRT-PCR confirmed the stumpy-enriched expression of this gene (Figure 4B), gene silencing via inducible RNAi in pleomorphic trypanosomes (Figure 5A) generated no reproducible effect on parasite growth, development to stumpy forms *in vivo* (Figure 5B) or in the kinetics of differentiation of stumpy forms to procyclic forms (Figure 5C). Since effective and inducible depletion of the target RNA was confirmed, this indicates that this gene was not important in the generation or differentiation of stumpy forms, showed redundancy with another protein, or may be involved in development in the tsetse fly.

### Analysis of genes that are Enriched in the Polysomal Fraction of Stumpy Forms

**Figure 6:**
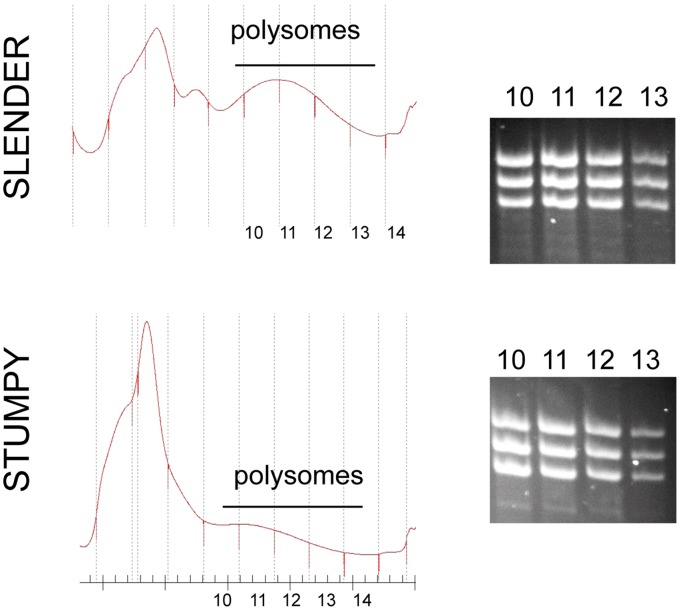
Polysome profiles of slender and stumpy T. brucei AnTat1.1. In each case the derived UV absorbance at 260nm was used to detect the polysomal fractions. Stumpy polysomal material was derived from a pool from 4 mouse infections, with RNA being isolated simultaneously and in parallel. Slender polysome material was isolated from six flasks of 50ml culture. RNA was prepared from fractions 10–13 and these were used to assay transcript levels via digital SAGE. RNA prepared from the polysome enriched fractions are shown to the right hand side in each case. These were pooled for Digital SAGE expression analysis.

To investigate transcripts that escaped the general translational repression observed in stumpy forms, total mRNA from slender and stumpy forms was subjected to polysome gradient fractionation in order to enrich actively translated mRNAs from each life cycle stage. Insufficient material was available to derive polysomal material from pleomorphic slender material, where cell numbers are required to be maintained at less than 3×10^7^/ml in infections. Hence, to achieve sufficient cell numbers, material was derived from monomorphic slender cells of *T. brucei* s427; these are a non-isogenic cell line grown in culture such that some expression differences may be related to strain or culture adaptation. For the stumpy material, total mRNA was isolated from a pooling of four replicate mouse infections. In each case material was fractionated on a 15–50% sucrose gradient; although the profile resolution was not sufficient to resolve discrete polysomal peaks the polysomal material was less abundant in the translationally quiescent stumpy forms than from slender forms, as expected [25] (Figure 6). This material was then pooled and processed for digital SAGE analysis and the abundance of transcripts compared between the total and polysomal material from the monomorphic slender and stumpy-derived samples using the methods previously described.

**Figure 7:**
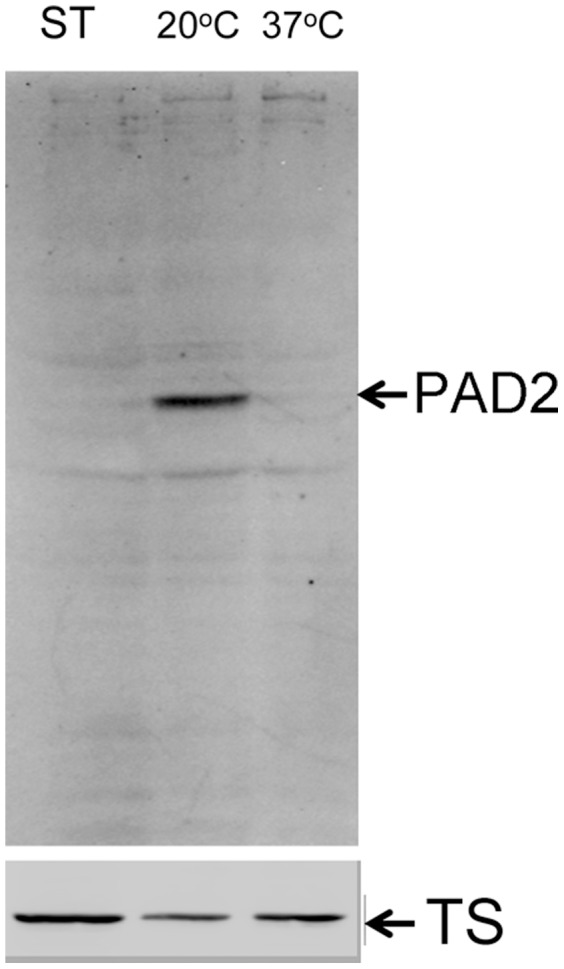
PAD2 is expressed predominantly only upon cold shock of stumpy forms. Western blot for the expression of PAD 2 in stumpy forms freshly harvested from a rodent infection (ST) or incubated in HMI9 medium at either 20°C or 37°C. PAD2 expression is clear at 20°C but little is expressed at 37°C. This matches the analysis previously reported by Dean et al 2009 [10]. Analysis of the same samples using an antibody to trypanothione synthetase (TS) provided a loading control.

**Figure 8:**
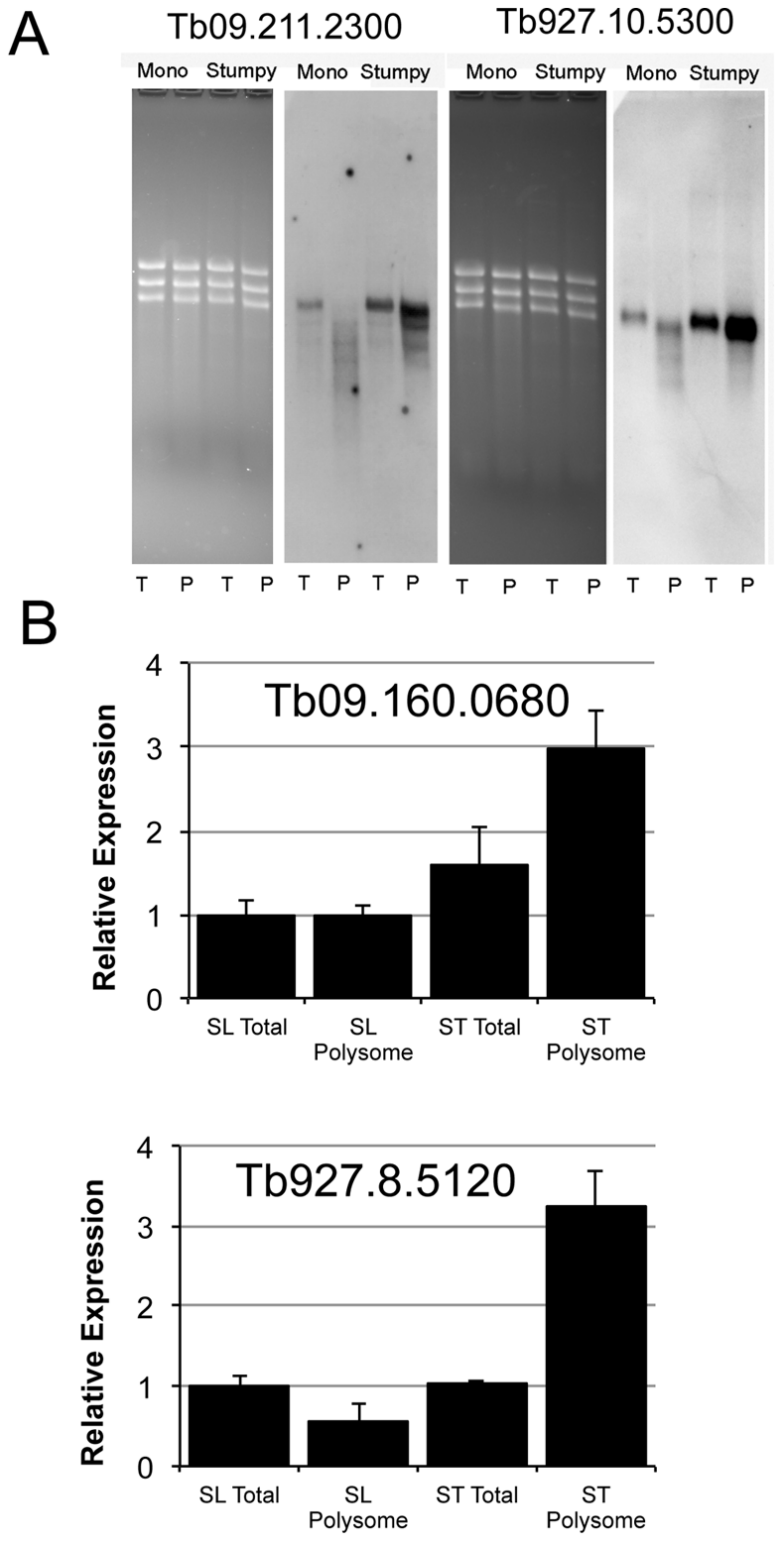
Validation of the polysomal enrichment of test mRNAs predicted from Digital SAGE analysis to exhibit elevated abundance in stumpy polysomal fractions. Total (T) and polysomal (P) mRNA mRNA derived from monomorphic slender and pleomorphic stumpy forms is shown, these being hybridized with riboprobes detecting *Tb*09.211.2300, or *Tb*927.8.5120. Both transcripts were confirmed as showing enrichment in stumpy form polysomal RNAs with respect to both stumpy total RNA and slender total and polysomal mRNA. Some smearing is evident in the polysomal material of slender and stumpy forms reflecting some non-specific cross reactivity with other transcripts; an absence of significant RNA degradation is evidenced by the integrity of rRNA bands on the ethidium bromide-stained formaldehyde gel. Realtime qRT PCR of two further transcripts provided confirmation of their respective enrichment in polysome-derived material.

Table 2 shows the 31 transcripts that were three-fold or greater enriched in the stumpy polysome fractions compared with the total stumpy mRNA profile (the full comparison is available in *Data File S6*). This represents mRNAs enriched to higher levels in large molecular weight RNP complexes and polysomes than expected from their overall mRNA abundance in the sample. Of these, the majority (15/31) were designated ‘hypothetical’, and several (9/31) had a predicted or known mitochondrial function, as expected in transmission adapted forms [40]. For non-mitochondrial proteins, heat shock and calpain-like cysteine peptidases were present. In contrast to total mRNA, only 2/31 of the polysome enriched stumpy transcripts show a differentiation phenotype in the previously published RITSeq targeted RNAi library. Conversely 13/31 show a significant defect in slender bloodstream survival if targeted by RNAi. This would suggest that while total mRNA of stumpy forms show a trend towards transcripts involved in transmission, polysome associated transcripts are more likely involved in cell maintenance and viability. Among the transcripts three fold or more depleted in the polysomal fraction compared to total RNA for stumpy forms there was a strong emphasis (103/212; 49%) on ribosomal proteins and proteins associated with ribosomal assembly, highlighting that the stumpy forms are quiescent and translationally repressed. In contrast, the large amount of ribosome associated transcript present in the total stumpy mRNA may enable translation to be rapidly re-activated when the differentiation signal is received in the tsetse mid-gut. PAD2 was also shown to be strongly depleted in the polysomal fractions despite its elevated abundance in total stumpy form mRNA, reflecting that this protein is not strongly translated in stumpy forms until exposure to cold shock and differentiation conditions [10] (Figure 7). Validating the enrichment of transcripts in the stumpy polysome material, Northern blots were used to assess two transcripts (irrespective of their enrichment P-value): eukaryotic translation initiation factor, eIF6 (*Tb*927.10.5300) and a putative ATP-dependent DEAD/H RNA helicase (*Tb*09.211.2300) (Figure 8A). An increased abundance of each transcript was observed in the polysomal material from stumpy forms, which is consistent with the elevated protein expression of eukaryotic translation initiation factor eIF6 in the transmissible life cycle stage (S. Monk and K. Matthews, in preparation). As well as Northern blotting, two transcripts *Tb*09.160.0680 (a Sec1 related protein) and *Tb*927.8.5120 (cytochrome C), identified as enriched in the stumpy polysome material by Digital SAGE, were confirmed to be enriched in the polysomal material by qRT PCR (Figure 8B).

**Table 2:** Transcripts enriched ≥3× in polysomal material compared to total mRNA in stumpy forms.

Gene ID	Stumpy Mean Expession	Stumpy Polysome Mean Expression	Log_2_ Fold Change	Product Description
*Tb*927.10.7020	2.25	27.54	3.61	acid phosphatase, putative
*Tb*927.10.470	2.25	22.21	3.30	choline dehydrogenase, putative
*Tb*927.10.9250	10.13	44.43	2.13	G-actin binding protein, putative,CAP/Srv2p, putative
*Tb*927.10.1400	14.63	58.64	2.00	hypoxanthine-guanine phosphoribosyltransferase (HGPRT)
*Tb*927.8.1220	637.01	2538.51	1.99	hypothetical protein, conserved (pseudogene)
*Tb*927.10.2980	33.76	123.50	1.87	proteasome regulatory non-ATPase subunit 11,19S proteasome regulatory subunit, Metallo-peptidase, Clan MP, Family M67 (RPN11)
*Tb*927.8.4480	52.90	184.81	1.80	hypothetical protein, conserved
*Tb*11.02.3230	76.53	266.56	1.80	hypothetical protein, conserved
*Tb*11.03.0250	262.23	900.07	1.78	cyclophilin a,cyclophilin type peptidyl-prolyl cis-trans isomerase (CYPA)
*Tb*927.10.11660	108.04	370.51	1.78	hypothetical protein, conserved
*Tb*927.10.10830	30.39	103.96	1.77	HNRNPA (HNRNPA)
*Tb*927.7.4070	1247.01	4248.03	1.77	calpain-like cysteine peptidase, putative,cysteine peptidase, Clan CA, family C2, putative
*Tb*927.10.11820	104.67	354.52	1.76	hypothetical protein, conserved
*Tb*11.02.3570	1188.49	3903.28	1.72	hypothetical protein, conserved
*Tb*927.7.2070	1556.52	5096.57	1.71	heat shock protein DNAJ, putative
*Tb*927.8.1750	276.86	878.75	1.67	hypothetical protein, conserved
*Tb*927.10.13710	477.20	1507.82	1.66	hypothetical protein, conserved
*Tb*927.1.1580	108.04	341.19	1.66	cytochrome c oxidase assembly factor, putative,electron transport protein SCO1/2, putative
*Tb*11.02.2860	175.57	550.88	1.65	hypothetical protein, conserved
*Tb*927.3.3780	140.68	439.82	1.64	tryparedoxin
*Tb*927.7.2780	3179.43	9918.59	1.64	hypothetical protein, conserved
*Tb*09.211.4513	2311.70	7205.04	1.64	kinetoplastid membrane protein KMP-11 (KMP-11)
*Tb*927.5.1030	522.21	1608.23	1.62	hypothetical protein, conserved
*Tb*927.7.4290	159.82	489.58	1.62	hypothetical protein, conserved
*Tb*927.7.1320	4025.78	12326.48	1.61	10 kDa heat shock protein, putative (HSP10)
*Tb*927.7.3710	3018.49	9156.23	1.60	hypothetical protein, conserved
*Tb*927.10.6080	621.26	1882.78	1.60	proteasome beta 5 subunit, putative,proteasome beta 5 subunit (PRCE)
*Tb*11.02.1290	534.59	1608.23	1.59	hypothetical protein, conserved
*Tb*11.01.7800	3127.66	9387.25	1.59	nucleoside diphosphate kinase (NDPK)

The abundance of transcripts in the stumpy polysomal fraction was also compared with the abundance transcripts in the monomorphic slender polysomal fraction (*Data File S7*). Although the data came from different parasite strains, we sought to identify transcripts preferentially enriched in stumpy form polysomes relative to slender forms: we identified 60 transcripts enriched >3 fold. Several genes involved in cell cycle control were enriched in the stumpy form polysomes compared to the proliferative slender form, including Cyclin 7 (*Tb*927.6.5020), UMSBP (*Tb*927.10.6060) and *Tb*NST (*Tb*11.02.0240). Conversely, some of the most significantly depleted genes in the stumpy polysomal material are those that would be expected to be needed for cell division and growth, such as β and α-tubulin, lipin and various histone and riboprotein complexes. Further analysis of this dataset involved comparison with the surface phylome analysis of slender and stumpy forms, to identify transcripts that were predicted to be membrane associated and polysomally enriched in stumpy forms, hence revealing molecules potentially important in transmission capacity (*Data File S*8). This subset included GRESAG4 family members, amino acid transporter family members, purine nucleoside transporters, ESAG3 pseudogenes and trans-sialidases.

## Discussion

The generation of bloodstream stumpy forms of *T. brucei* is associated with cell cycle arrest and translational repression as the parasites await uptake in a tsetse blood meal. They also show pre-adaptation for transmission, including the partial activation of their mitochondrion [41], [42], such that upon transmission they can rapidly undergo the developmental changes necessary for colonisation of the tsetse fly mid-gut. Here, we have described the progression from translational arrest to translational reactivation during differentiation to procyclic forms, demonstrating that this occurs around 4–6 hours after differentiation is triggered with cis-aconitate. Moreover, we analysed the transcriptome of intermediate and stumpy forms from two different strains in order to identify the consistent adaptations that accompany development of the parasite’s transmission stage. Finally, we have analysed the transcripts that associate with polysomal material, reflecting transcripts that either escape translational silencing in stumpy forms (and hence may be important at this life cycle stage for survival or transmission) or those associated with high molecular weight RNP complexes, potentially regulating their expression positively or negatively. This provides a useful complement to direct analyses of protein synthesis via approaches such SILAC labelling, for example [43], [44]. Indeed these studies provide different read outs, one being a snapshot of mRNAs associating with polysomal fractions in the stumpy form, the other revealing proteins actively made in slender or procyclic forms [43], [44], with proteins synthesised in stumpy forms being inferred by comparison of unlabelled peptides against those in the labelled sample [44]. Since this does not involve the direct labelling of synthesised proteins in stumpy forms, proteins made throughout the transition from intermediate to stumpy forms would be identified, as well as those actively made in only stumpy forms, whereas procyclic proteins already synthesised in stumpy forms might be underrepresented. Hence SILAC might over-assign proteins translated in stumpy forms, whereas polysome analysis of stumpy forms would contain actively translated transcripts, but also transcripts associated with ribonucleoprotein complexes that co-sediment with translating ribosomes. In combination these different approaches are valuable and complementary, therefore.

Several publications have described the transcriptome and proteome profile of the different developmental stages of kinetoplastid parasites [45], [46], [47], [48]. In *T. brucei,* studies have largely focused on parasites cultured *in vitro* [27], [30] although three have analysed parasites harvested from infections [17], [28], [40]. These have been based on glass slide microarrays, Nimblegen arrays and, more recently, quantitatively more powerful next generation sequencing transcriptome studies. Of the latter, only one study has analysed by RNAseq approaches the expression profile of pleomorphic slender and stumpy forms derived from rodent infections [49]. These data are more representative of parasites in the field but were limited in biological sample number, such that material was analysed from single slender and stumpy RNA preparations. Hence, these are potentially subject to non-systematic variations resulting from growth in vivo, the given strain analysed and parasite isolation procedures. This variation is apparent when comparing the genes enriched in stumpy cells in different analyses with those enriched in stumpy cells in our study, although we found the best agreement (p<0.001, using a Monte Carlo simulation using 1000 iterations [50]) with the study by Jensen and colleagues [17] (*Data File S9*). The importance of different isolation procedures for different studies is revealed in the SILAC analysis of stumpy forms where PAD2 was seen to be strongly expressed, likely reflective of parasite cold shock or the induction of early differentiation events during isolation procedures [44].

In the study described here we have used Digital SAGE (based on its lower cost and so potential for the analysis of more biological samples) and analysed RNA preparations from two distinct strains of pleomorphic trypanosomes, and included RNA from intermediate forms in addition to slender and stumpy forms. This provides useful information because (i) consistent expression changes associated with the developmental form rather than parasite strain can be identified and (ii) the trends in expression during the transformation from slender to stumpy forms via intermediate forms provides a reassurance for transcripts whose expression becomes progressively increased or decreased during the transition to transmissible stages. Both components add value to the dataset, allowing genes whose expression fluctuates dramatically under different conditions (potentially unrelated to development) to be identified as well as transcripts elevated early in the development to transmission stages.

Matching earlier studies [44], [49], mitochondrial transcripts and proteins were among those most elevated during development to stumpy forms. However, Gene Ontology (GO) terms associated with translation, ribonuclear complexes and macromolecular biosynthetic processes were also enriched, emphasising the preadaptation of stumpy forms to onward development in the tsetse fly. This was further supported by analyses of the enriched metabolic pathways in stumpy forms, where there was the expected up regulation of TCA cycle components, oxidative phosphorylation, amino acid metabolism as well as components associated with ribosomal components. Glycolysis was down regulated. The dataset of enriched transcripts was also analysed with respect to their surface phylome categories [38] in order to identify membrane-associated molecules potentially up-regulated in stumpy forms, and of importance for transmission or stumpy form viability. This highlighted the up-regulation of amino acid transporters, purine nucleoside transporters (including the previously characterised NT10, enriched in stumpy and procyclic forms [35]), and surface carboxylate transporters of the PAD family. Additionally, two magnesium transporter proteins of the CorA type Mgt2 family were identified in the stumpy enriched set. These proteins have been previously characterised in *Leishmania* parasites [39] and proposed to have ER association. Moreover, the Mgt proteins show developmental regulation, with Mgt2 in particular affecting parasite virulence and development from promastigotes to amastigotes. Additionally, an mRNA encoding a protein with a predicted CRAL-TRIO domain was identified among the transcripts significantly enriched in stumpy forms. Further analysis of this molecule confirmed its developmental regulation between slender and stumpy forms by qRT PCR and Northern blotting. However, RNAi mediated knock down of this transcript in pleomorphic parasites did not reveal a detectable phenotype during development from slender to stumpy forms *in vivo*, nor during differentiation to procyclic forms *in vitro*. Hence this molecule represents a candidate with a role for establishment in the tsetse fly, rather than the differentiation process *per se*. A similar observation would be expected with another of the enriched phylome categories, namely trans-sialidases. These scavenge sialic acid from the blood-meal of the tsetse fly and protect the parasite from the insect’s midgut environment, aiding parasite transmission [51].

Polysomal associated material was also analysed in stumpy forms to assist the identification of transcripts that might escape the overall translational repression characteristic of this transmission stage. However, transcripts associated with larger ribonuclear complexes that could play a positive or negative role in the expression of the associated mRNAs (e.g. transcripts poised for translation but not actively translated; stress granule or P body complexes) might also be identified by this approach. Nonetheless, the measured global translational quiescence of stumpy forms was also emphasised by the depletion of mRNAs encoding ribosomal proteins in the stumpy polysomal material. Moreover, comparison of the proteins preferentially expressed in stumpy forms and the transcripts enriched in the stumpy polysomal material with respect to slender polysomal material showed a higher degree of overlap with SILAC proteome studies then would be expected by chance (assessed using a Monte Carlo simulation of the distribution, p value<0.001 (*Data File S9*)). When analysed for regulatory motifs by the Regulatory Sequences Analysis Tool (RSAT) [52], the 3′UTRs of transcripts that were polysome-enriched in either slender or stumpy forms revealed that the motif TTATT was over-represented in polysomal stumpy transcripts (*Data File S10*). A similar motif was also identified when analysing procyclic enriched transcripts in a previous study by the same approach [53].

### Conclusions

The comparison between different strains of trypanosomes revealed that developmental responses at the mRNA level were sufficiently similar to be considered biological replicates of the same material. Moreover, comparison of the transcriptome datasets revealed good agreement for those transcripts previously highlighted as being up-regulated in either slender or stumpy forms based on Nimblegen analysis in another trypanosome strain, *T. brucei* TREU927/4 [17]. Thus the biological events that trypanosomes undergo as they prepare for transmission are quite predictable and consistent regardless of strain, reflective of an underlying developmental pathway. By identifying the genes that are controlled in slender, intermediate and stumpy forms in the blood, genes that are coincidently, or differentially regulated can be identified. This assists an analysis of the temporal mRNA hierarchy that bloodstream trypanosomes employ as they perceive and respond to the parasite-derived density-sensing signal as an adaptation for transmission. Analyses of the transcripts that cosediment with polysomes also reveals transcripts actively translated, as well as those associated with high molecular weight RNP complexes. Integration of this information with studies of the proteins present at each life cycle stage by proteome studies will assist understanding of how trypanosomes prepare for transmission through holding transcripts poised for translation as well as actively translating transcripts when the bulk of mRNAs are translationally quiescent.

## Materials and Methods

### Ethics Statement

Animal experiments in this work were carried out in accordance with, and approved by, the Ethical Review Committee of the University of Edinburgh and were carried out under a project licence (60/4373) issued under the UK Home Office Animal (Scientific Procedures) Act (1986).

### Trypanosomes

*Trypanosoma brucei bru*cei AnTat1.1 was used for the derivation of material from slender, intermediate and stumpy forms, transcriptome analysis and validation by qRT PCR and Northern blotting. Slender, intermediate and stumpy form material was also derived from an EATRO (East African Trypanosomiasis Research Organisation) strain of *Trypanosoma brucei brucei*. In each case slender material was derived on Day 3 post infection, intermediate material from day 4–5 and stumpy material from day 6–7, with morphology being used in each case to identify the relevant developmental stages. Parasites were harvested from mouse (female MF1) infections and maintained at 37°C prior to DEAE column purification, also at 37°C. For monomorphic bloodstream form mRNA analysis and for procyclic form analyses, *T. brucei brucei* Lister S427 was used. For transfection of pleomorphic trypanosomes, *T. brucei* AnTat1.1 90:13 was used, these being a kind gift of Profs Michael Boshart and Markus Engstler, transfection and selection being carried out according to [54].

### Polysome Analysis and RNA Purification

For the bloodstream monomorphic cell samples, cells were grown in 5 flasks of 50 ml culture volumes to a cell density of 1–2×10^6^ cells/ml. The cells were incubated with cycloheximide (100 µg/ml) for 10 minutes at 37°C, then pelleted by centrifugation for 10 minutes, pooled, and and washed twice in ice-cold PSG containing 100 µg/ml cycloheximide. For the stumpy cell sample, *T. brucei* AnTat1.1 cells were purified from the blood of four mice 6 days post-infection, cell density was determined and then the cells were incubated with cycloheximide (100 µg/ml) for 10 minutes at 37°C. After centrifugation for 10 minutes the cells were washed once with ice-cold PSG containing 100 µg/ml cycloheximide. Samples were then stored at −80°C. Next, 50% and 15% sucrose solutions (w/v; sucrose in polysome buffer) were sterilized through a syringe filter (0.22 µm; Sartorius Stedim Biotech) and completed with 100 µg/ml cycloheximide, 0.5 µl/ml RNase inhibitor (RNasin, Promega), protease inhibitor cocktail (1 tablet per 50 ml polysome buffer; Roche) and 0.1mM DTT. Gradients were poured using a Hoefer SG 50 Gradient Maker (Amersham Biosciences), magnetic stirrer and MasterFlex C/L peristaltic pump (Cole-Parmer) in to Polyallomer centrifuge tubes (14×95 mm; Beckman) and stored at briefly (<2 h) at 4° until ultra-centrifugation and fractionation. Cell samples were lysed (unthawed) through sonication for ∼3–6 minutes in a water bath with sonication (Bandelin Sonorex) with brief periods of vortexing. 5 µl of sample was removed to confirm cell lysis by microscopic examination. Cell lysates were then centrifuged at 12 000 rpm for 4 minutes at 4°C and the supernatant transferred to a fresh eppendorf tube containing 1 µl of RNase inhibitor (RNasin). Cell lysate was then layered on to the top of a 15–50% sucrose gradient and centrifuged in a pre-cooled Beckman L-60 Ultracentrifuge at 40 000 rpm for 2 hours at 4°C, using a SW40 rotor. Thereafter, the centrifuge tube was pierced with a Brandel Tube Piercer and Fluorinert FC-77 (Sigma-Aldrich) pumped through as the high-density chase solution with a Brandel Syringe Pump. The gradient was then separated in to fractions using a Foxy R1 fractionator (Teledyne Isco) (one fraction was taken for every 13 drops) and the optical density read at 254 nm throughout fractionation on a UA-6 Absorbance Detector (Teledyne Isco). PeakTrak software (Teledyne Isco, Inc.) was used to record the absorbance output.

RNA extraction was carried out using the Qiagen RNeasy kit. Following fractionation, 3.5 ml of Buffer RLT with β-mercaptoethanol (10 μl β-mercaptoethanol per 1 ml RLT buffer) was added to each polysome fraction. After overnight storage at −80°C, 2.5 ml of 100% ethanol was added and mixed. RNA was then extracted from each fraction sample using a separate RNeasy column and according to the manufacturer’s instructions for the ‘RNA cleanup’ protocol, including on-column DNase treatment (RNase-Free DNase Set, Qiagen). RNA was eluted from each column with 30 or 40 μl nuclease-free water and stored at −80°C. RNA concentration was determined using a NanoDrop Spectrophotometer ND-1000 and for the Illumina digital tag sequencing experiment, 10% of each sample was examined on an RNA gel to confirm RNA integrity.

### RNA Analysis and Differentiation Analysis

Northern blotting was carried out according to [29]; RT-PCR was carried out according to [55]. Growth assays for parasitaemia in mice were carried out using the rapid matching analysis of [56]. Differentiation assays were carried out according to [8]. Analyses of differentiation to procyclic forms were carried out using anti-EP procyclin monoclonal antibody (Cedarlane Laboratories, Canada) diluted 1∶500, as described in [8].

### Data Submission

The data discussed in this publication have been deposited in NCBI's Gene Expression Omnibus and are accessible through GEO Series accession number GSE46388 (http://www.ncbi.nlm.nih.gov/geo/query/acc.cgi?acc=GSE46388).

### [^35^S]-methionine Labelling

The radiolabelled methionine experiments were performed similarly to previously reported [28], [57]. Cells were pelleted, washed with methionine-free minimal essential media and resuspended with 2 ml of the same media to a concentration of 1×10^7^ cells/ml. Cells were labelled for one hour at 37°C with 10 µCi [^35^S]-methionine in an orbital shaker water bath.

For use with SDS-PAGE, a volume of 1 ml was pelleted, washed with trypanosome dilution buffer (TDB; 25 mM KCl, 400 mM NaCl, 5 mM MgSO_4_, 100 mM Na_2_HPO_4_, NaH_2_PO_4_, 100 mM glucose), suspended with pre-heated SDS-PAGE sample buffer and boiled for 5 minutes. A 10% SDS-PAGE gel and Coomassie blue staining was used to visualise the proteins. The gel was then destained, soaked in En^3^hance (NEN) for 30 minutes, washed twice with water, soaked in 10% glycerol, dried and exposed to XAR-5 film at −70°C.

For radiolabel incorporation quantification, the remaining 1 ml was split in to triplicate samples, these being centrifuged, washed with TDB, then resuspended with TCA. The protein pellet was washed and reconstituted with 1% SDS, the incorporation quantified scintillation counting.

## Supporting Information

Table S1 Number of total tag sequences derived from the respective experimental analyses and the number of aligned tags from each to the genome.
(DOCX)

Data File S1 **Overview of relative RNA abundance of genes compared for each life-cycle stage.**
(XLSX)

Data File S2 **Genes that show significant (p<0.1) RNA abundance differences between slender and stumpy life-cycle stages.** Red indicates strong up-regulation in stumpy forms and blue indicates strong down-regulation.
(XLSX)

Data File S3 **Genes that show significant (p<0.1) RNA abundance differences between slender and intermediate life-cycle stages.** Red indicates strong up-regulation in intermediate forms and blue indicates strong down-regulation.
(XLSX)

Data File S4 **The relative RNA abundance of genes in known metabolic pathways compared between the various stages life-cycle stages.**
(XLS)

Data File S5 **The surface phylome categories for genes that show strong up-regulation of RNA abundance between slender and stumpy life-cycle stages (surface phylome categories sourced from** [**38**] **).**
(XLSX)

Data File S6 **Relative RNA abundance of genes comparing the polysomal and total RNA fraction of stumpy cells.** Genes that show significant (p<0.1) RNA abundance differences are coloured, with red indicating strong up-regulation in the polysomal fraction and blue indicating strong down-regulation in the polysomal fraction.
(XLSX)

Data File S7 **Relative RNA abundance of genes comparing the slender and stumpy form polysomal fractions.** Genes that show significant (p<0.1) RNA abundance differences are coloured, with red indicating strong up-regulation in the stumpy polysomal fraction and blue indicating strong down-regulation in the stumpy polysomal fraction.
(XLSX)

Data File S8 **The surface phylome categories for genes that show higher RNA abundance in the stumpy polysomal fraction compared to the slender polysomal fraction (surface phylome categories sourced from** [**38**] **).**
(XLSX)

Data File S9 **Comparison between transcripts enriched in stumpy forms in this study with earlier published studies.**
(DOCX)

Data File S10 **Analysis of the enrichment of oligonucleotide motifs in the 3′UTR of transcripts enriched in slender or stumpy polysomal material.**
(DOCX)
