# Supplementary material for: Regulation of Trypanosoma brucei Total and Polysomal mRNA during Development within Its Mammalian Host
Source: PLoS One. 2013 Jun 26;8(6):e67069. doi: 10.1371/journal.pone.0067069 (PMC3694164; doi:10.1371/journal.pone.0067069)
Supplement: Table S1 — Number of total tag sequences derived from the respective experimental analyses and the number of aligned tags from each to the genome. (DOCX) [file pone.0067069.s001.docx]

**Table S1**

| **Sample** | **Total Tags** | **Aligned Tags** | **Genes With Tags** |
| --- | --- | --- | --- |
| AnTat_SL | 13082843 | 6205609(47.4%) | 8744 |
| AnTat_INT | 14137741 | 7289670 (51.6%) | 8666 |
| AnTat_ST | 9628929 | 4994423 (51.9%) | 8621 |
| AnTat_ST_Poly | 10909487 | 5571275 (51.1%) | 8577 |
| EATRO_SL | 3646329 | 1721784 (47.2%) | 8007 |
| EATRO_INT | 3699620 | 2028054 (54.8%) | 8049 |
| EATRO_ST | 2635955 | 1490630 (56.6%) | 8121 |
| L427_SL | 3420164 | 1644934 (48.1%) | 8563 |
| L427_SL_Poly | 9731186 | 4835767 (49.7%) | 8659 |
