# Supplementary material for: Regulation of Trypanosoma brucei Total and Polysomal mRNA during Development within Its Mammalian Host
Source: PLoS One. 2013 Jun 26;8(6):e67069. doi: 10.1371/journal.pone.0067069 (PMC3694164; doi:10.1371/journal.pone.0067069)
Supplement: Data File S9 — Comparison between transcripts enriched in stumpy forms in this study with earlier published studies. (DOCX) [file pone.0067069.s010.docx]

**Supplementary Data 9**

Analysis of the agreement between different transcriptome datasets in the published literature and the analysis presented in this manuscript. In each case, a Monte Carlo simulation was used with 1000 iterations to determine the commonality between stumpy enriched transcripts in the published study (group1, red dots) and this study (group 2, grey dots), with a null hypothesis that the overlap between the datasets occurs by chance.

A. Analysis vs. Jensen et al. (P<0.001) B. Analysis vs. Queiroz et al. (p=0.042)


C. Analysis vs. Kabani et al 2009 (p=0.068) D. ST polysome enriched (with respect to SL polysome enriched) vs. SILAC proteome (Gunasekera et al., 2012) (p<0.001)
