# Supplementary material for: Regulation of Trypanosoma brucei Total and Polysomal mRNA during Development within Its Mammalian Host
Source: PLoS One. 2013 Jun 26;8(6):e67069. doi: 10.1371/journal.pone.0067069 (PMC3694164; doi:10.1371/journal.pone.0067069)
Supplement: Data File S10 — Analysis of the enrichment of oligonucleotide motifs in the 3′UTR of transcripts enriched in slender or stumpy polysomal material. (DOCX) [file pone.0067069.s011.docx]

**Supplementary Data 10**

Oligomer (8-mer) sequences, found by Regulatory Sequences Analysis Tool (RSAT; http://rsat.scmbb.ulb.ac.be/)[[1](#_ENREF_1)] oligo-analysis tool, over-represented in either 300nt of downstream-sequences of stumpy form or slender form polysomal transcripts^a^.

^a^ Using RSAT oligo-analysis, a background model of oligomer (8-mer) sequence occurrence frequency was created from 300nt downstream-sequences for genes on Chromosomes 1 and 2. This was used to identify over-represented 8-mers in the 300nt downstream-sequences of genes enriched in either stumpy form or slender form polysome-associated RNA. The over-represented 8-mers were then compared to find unique 8-mers present in each dataset.

1. van Helden J, Andre B, Collado-Vides J (1998) Extracting regulatory sites from the upstream region of yeast genes by computational analysis of oligonucleotide frequencies. J Mol Biol 281: 827-842.
